# Newly Diagnosed Crohn’s Disease After SARS‐CoV‐2 Infection

**DOI:** 10.1155/crgm/2252518

**Published:** 2026-01-31

**Authors:** Hironori Yamada, Toru Yamada, Shuji Ouchi, Ryota Nakamura, Akiko Tamura, Iichiroh Onishi, Masayoshi Hashimoto

**Affiliations:** ^1^ Department of General Medicine, Graduate School of Medical and Dental Sciences, Institute of Science Tokyo, Tokyo, Japan; ^2^ Department of Gastroenterology and Hepatology, Graduate School of Medical and Dental Sciences, Institute of Science Tokyo, Tokyo, Japan; ^3^ Division of Pathology, Graduate School of Medical and Dental Sciences, Institute of Science Tokyo, Tokyo, Japan

## Abstract

Coronavirus disease 2019 (COVID‐19) is known to induce gastrointestinal symptoms as well as respiratory symptoms. There have been instances where diarrhea persists after the acute phase of COVID‐19, suggesting an extension of the disease’s symptoms. It is not typical to recall the onset of inflammatory bowel disease (IBD) with such symptoms, much less with reports on Crohn’s disease (CD). It is important to collect such cases in order to lead to appropriate diagnosis and treatment. This report presents a case of a young man in which diarrhea, initially manifesting during the acute phase of COVID‐19, persisted for two months, ultimately leading to a CD diagnosis. At the onset of COVID‐19, the patient had fever, abdominal pain, and diarrhea, but respiratory symptoms were not prominent. An ileocolonoscopy was performed to further investigate the cause of persistent diarrhea, leading to an appropriate diagnosis of CD. It is hypothesized that aberrations in the immune system triggered by severe acute respiratory syndrome coronavirus 2’s impact on the intestinal tract might contribute to the onset of CD. The patient’s condition gradually improved after the initiation of treatment with prednisolone. By the following treatment with azathioprine, the patient has maintained clinical remission. Clinicians should consider performing ileocolonoscopy for patients with persistent diarrhea after COVID‐19, given the possibility of IBD. Gastrointestinal symptoms are relatively common with COVID‐19. COVID‐19 infection may trigger CD through immunological mechanisms. It is important to consider that cases of prolonged diarrhea after COVID‐19 may include the induction of CD and to perform an ileocolonoscopy.

## 1. Introduction

Crohn’s disease (CD) is a type of inflammatory bowel disease (IBD). The incidence of CD varies by country and has shown an annual increase of 4%–11% [1]. Factors contributing to CD onset include genetic predispositions, environmental factors, microbial elements, changes in gut microbiota, and immune regulation disorders [2]. However, the role of viral infections as a trigger for CD onset remains unclear, and its immunologic mechanisms may differ from virus to virus. Gastrointestinal symptoms such as diarrhea and abdominal pain are increasingly recognized as part of both the acute and postacute spectrum of Coronavirus disease 2019 (COVID‐19). Follow‐up studies have reported that around 10% of patients experience persistent gastrointestinal complaints, including chronic diarrhea, several months after infection [3, 4]. Although reports of CD onset specifically triggered by COVID‐19 remain limited, distinguishing de novo CD from these common post‐COVID‐19 sequelae is challenging. Delayed diagnosis of CD can lead to progressive bowel damage with stricturing or penetrating complications, malnutrition, and impaired growth, especially in adolescents and young adults [2]. Therefore, recognizing atypical presentations and triggers is crucial for timely diagnosis and treatment. This report presents a case of an 18‐year‐old man who developed persistent watery diarrhea for 2 months following COVID‐19 infection, ultimately leading to a CD diagnosis.

## 2. Case Presentation

An 18‐year‐old Indian man presented to the general clinic with a complaint of diarrhea for 2 months following COVID‐19. He had been generally healthy, but two months before presentation, he developed acute intermittent abdominal pain, watery diarrhea, and fever and was confirmed as positive for severe acute respiratory syndrome coronavirus 2 (SARS‐CoV‐2) via polymerase chain reaction (PCR) testing at another clinic. He received outpatient treatment for COVID‐19. However, despite treatment, he continued to experience low‐grade fever (approximately 37°C) and watery diarrhea 2‐3 times daily. One month after the onset of diarrhea, he visited another hospital. Although a PCR test for SARS‐CoV‐2 was negative at this time, he was diagnosed with prolonged COVID‐19 symptoms and placed under observation. His symptoms persisted and his weight dropped 8 kg in 2 months, leading to referral to our outpatient department, 2 months after symptom onset.

His medical history comprised only seasonal allergies, and his only regular medication was levocetirizine. The patient denied any family history of malignant tumors and autoimmune disorders. At the time of presentation in 2023, the patient had no history of smoking, alcohol use, or illicit drug use.

On examination, the patient reported intermittent abdominal pain lasting 2 months, watery diarrhea 2–3 times per day, and fever of approximately 37°C. Physical examination showed clear consciousness, no abdominal tenderness, normal bowel sounds, and a body temperature of 37.4°C. All other vital signs were within normal ranges.

The detailed laboratory results at the first presentation to our hospital are summarized in Table 1. A full blood count showed a normal white blood cell count of 7100/μL. Serum biochemistry tests revealed low serum albumin (2.9 g/dL) and elevated C‐reactive protein concentration (5.93 mg/dL). Fecal calprotectin, measured by the OC‐SENSOR PLEDIA analyzer (Eiken Chemical Co., Ltd., Tokyo, Japan), which employs a latex agglutination turbidimetric immunoassay, was markedly elevated (> 6000 μg/g). Renal and liver function tests and electrolyte concentrations were within normal ranges. PCR testing performed at our hospital using the Xpert Xpress SARS‐CoV‐2 kit (Cepheid, USA/Sweden) on a nasopharyngeal swab sample was negative for SARS‐CoV‐2. Note that the specific kit used for the initial positive diagnosis at the previous clinic is unavailable.

**TABLE 1:** Laboratory data on admission.

Parameter	Result
*Hematology*	
WBC (/μL)	7100
Neutrophils (%)	54.6
Lymphocytes (%)	33.5
Monocytes (%)	10.0
Eosinophils (%)	1.6
Basophils (%)	0.3
RBC (10^4^/μL)	486
Hb (g/dL)	11.0
Hct (%)	37.3
MCV (fL)	76.7
MCH (pg)	22.6
Plt (10^4^/μL)	44.3
ESR (mm/hr)	64

*Blood gas analysis*	
pH	7.441
pCO_2_ (mmHg)	36.8
pO_2_ (mmHg)	115
HCO_3_^-^ (mmol/L)	24.6
Lactate (mmol/L)	0.5

*Coagulation*	
PT (%)	78.7
PT‐INR	1.12
APTT (sec)	32.4
D‐dimer (μg/mL)	0.5

*Biochemistry*	
TP (g/dL)	6.8
Alb (g/dL)	2.9
AST (U/L)	11
ALT (U/L)	15
LDH (U/L)	105
γ‐GTP (U/L)	43
CK (U/L)	26
Amylase (U/L)	114
T‐Bil (mg/dL)	0.4
BUN (mg/dL)	8.5
Cre (mg/dL)	0.71
Glucose (mg/dL)	68
T‐Chol (mg/dL)	158
TG (mg/dL)	127
HDL‐Chol (mg/dL)	44
Na (mEq/L)	139
K (mEq/L)	4.4
Cl (mEq/L)	102
Ca (mg/dL)	9.1
IP (mg/dL)	4.1
Mg (mg/dL)	2.0
Fe (μg/dL)	13
UIBC (μg/dL)	218
Ferritin (ng/dL)	198
Vitamin B_12_ (pg/mL)	193
Folate (ng/mL)	5.8

*Serology and immunology*	
CRP (mg/dL)	5.93
IgG (mg/dL)	1397
IgA (mg/dL)	196
IgM (mg/dL)	117
ANA	× 40
MPO‐ANCA (EU)	1.0
PR3‐ANCA (EU)	1.0
Fecal calprotectin (μg/g)	> 6000

*Infection*	
HIV Ab	—
Syphilis (RPR/TPAb)	—
HBs Ag (IU/mL)	—
HCV Ab	—

*Endocrinology*	
TSH (μIU/mL)	1.38
FT3 (pg/mL)	1.52
FT4 (ng/dL)	1.03

*Urine analysis*	
Specific gravity	1.016
pH	5.5
Protein	—
Glucose	—
Ketone bodies	—
WBC (/HPF)	—
RBC (/HPF)	—

*Note:* Hb, hemoglobin; Hct, hematocrit; Plt, platelets; Alb, albumin; AST, aspartate aminotransferase; ALT, alanine aminotransferase; LDH, lactate dehydrogenase; γ‐GTP, gamma‐glutamyl transpeptidase; T‐Bil, total bilirubin; Cre, creatinine; T‐Chol, total cholesterol; TG, triglycerides; HDL‐C, high‐density lipoprotein cholesterol; CRP, C‐reactive protein; Ig, immunoglobulin; ANA, antinuclear antibody; ANCA, antineutrophil cytoplasmic antibody; TSH, thyroid‐stimulating hormone; FT3, free triiodothyronine; FT4, free thyroxine; HPF, high power field.

Abbreviations: APTT = activated partial thromboplastin time, BUN = blood urea nitrogen, CK = creatine kinase, ESR = erythrocyte sedimentation rate, IP = inorganic phosphorus, MCH = mean corpuscular hemoglobin, MCV = mean corpuscular volume, PT = prothrombin time, RBC = red blood cells, TP = total protein, WBC = white blood cells.

Contrast‐enhanced abdominal computed tomography revealed edematous thickening of the bowel wall from the distal ileum to the terminal ileum, but no caliber change suggesting an obstruction was observed. Edema was also suspected in the proximal ascending colon. There was a small amount of ascites but no lymph node enlargement, with no other specific findings in the abdomen.

Upper gastrointestinal endoscopy revealed reflux esophagitis (Grade M) and atrophic gastritis. During ileocolonoscopy, multiple longitudinal ulcers and cobblestone pattern were observed in the terminal ileum (Figure 1(a)). Multiple longitudinal ulcers were observed from the cecum to the ascending colon. The intervening mucosa appeared relatively clean, with no prominent erythema or edema (Figures 1(b) and 1(c)). In the descending colon, multiple erosions with mucus deposition were observed (Figure 1(d)). There was scattered mucosal erythema and small erosions from the terminal ileum to the ascending colon, with no anal lesions noted.

FIGURE 1Ileocolonoscopy: (a) terminal ileum, (b) cecum, (c) ascending colon, and (d) descending colon.

**Figure:**
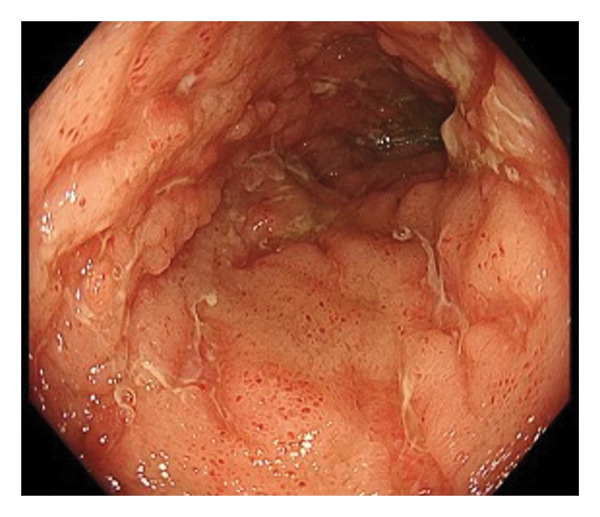
(a)

**Figure:**
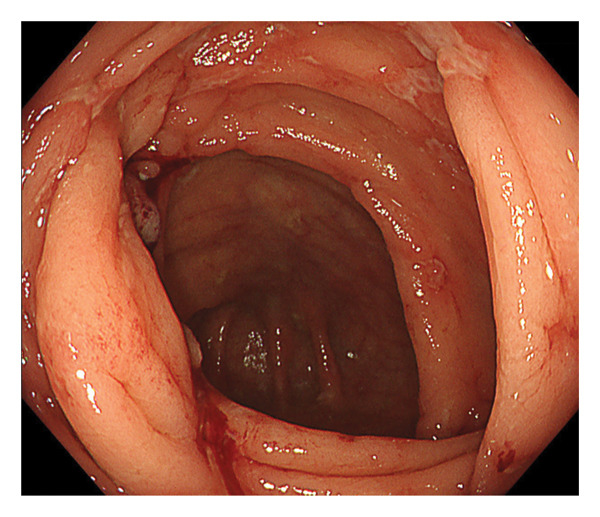
(b)

**Figure:**
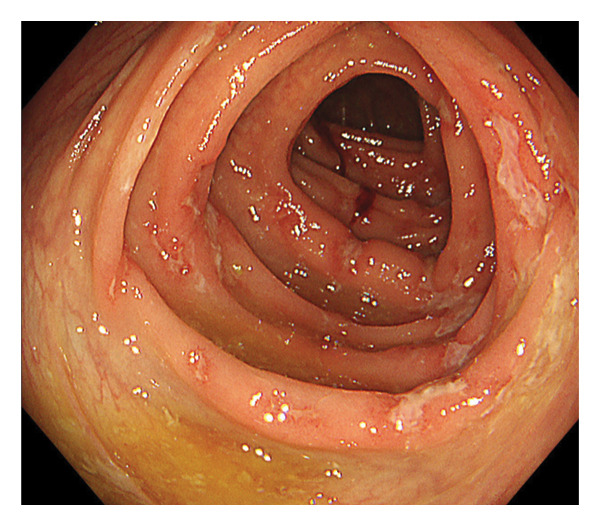
(c)

**Figure:**
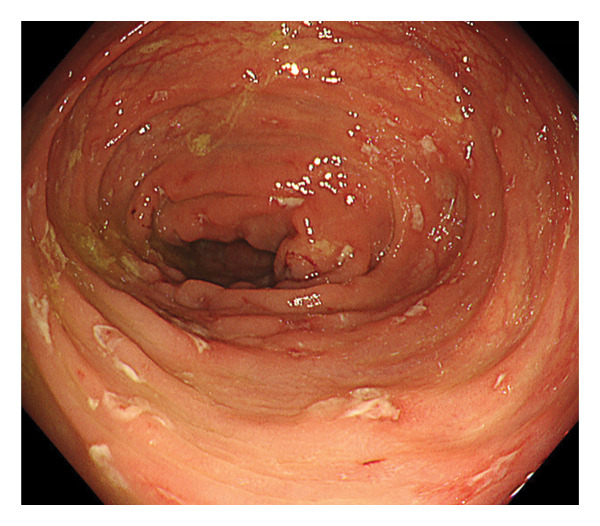
(d)

**FIGURE 2:**
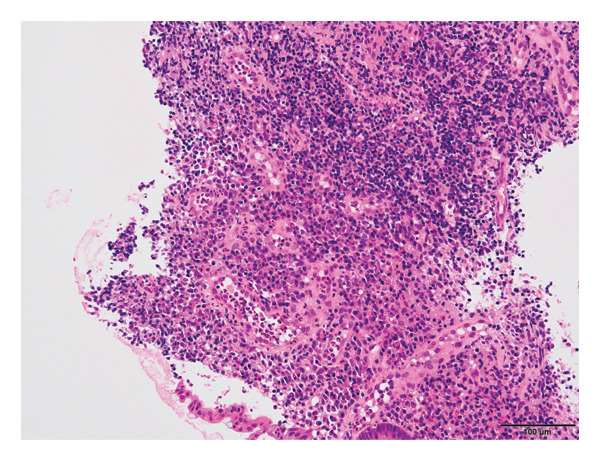
Histopathology of the ascending colonic biopsy. Hematoxylin and eosin staining (× 200).

Histopathological examination from biopsies of the ileum and colon revealed preserved villous architecture in the ileal mucosa with infiltration of plasma cells and eosinophils in the interstitium. In the ascending colonic mucosa, there was notable infiltration of plasma cells and lymphocytes, with granulation tissue and regenerative changes in the epithelium (Figure 2). No granulomas, amoebae, or inclusion bodies were observed. Given the observation of a longitudinal ulcer and cobblestone appearance, a definitive diagnosis of CD was established.

Magnetic resonance enterocolonography showed wall thickening, intramural edema, and limited diffusion from the distal ileum to the terminal ileum, with ulceration and fat stranding (Figure 3). There was no dilatation of the oral bowel or wall thickening of the jejunum. The wall of the proximal ascending colon was slightly thickened, with intramural edema and high signal on diffusion‐weighted images. The descending colon and sigmoid colon also had thick walls and high signal on diffusion‐weighted images, which could have been due to collapse. There were no abnormalities in the rectum or perianal region.

FIGURE 3Magnetic resonance enterocolonography of (a) overall view and (b) the ascending colonic region.

**Figure:**
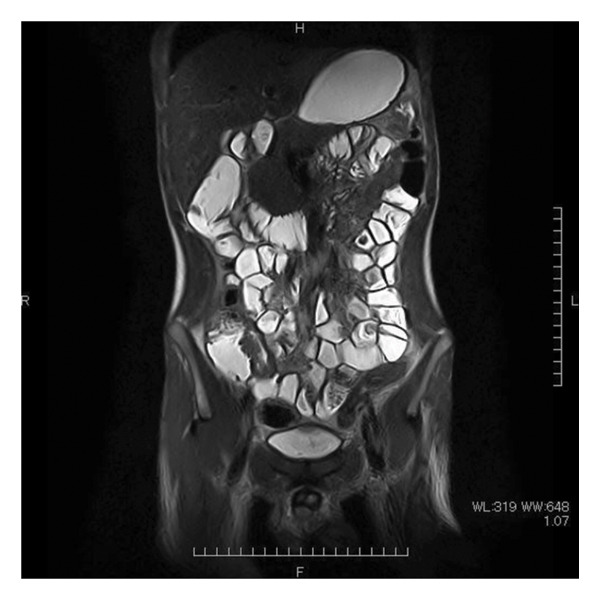
(a)

**Figure:**
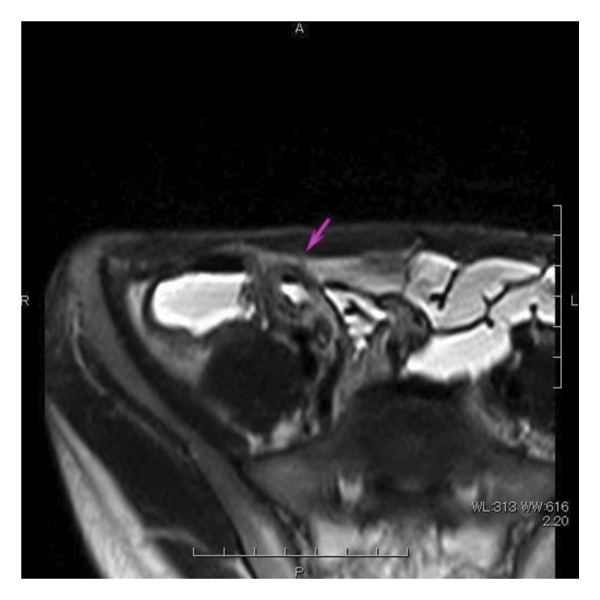
(b)

## 3. Further Diagnostic Workup

On the basis of chronic watery diarrhea together with the endoscopic and histopathological findings, the patient was diagnosed with CD. The submitted stool sample was sent for bacterial culture, which showed no significant pathogenic growth. Although specific testing for ova and parasites (including *Entamoeba histolytica* and *Giardia* species) and viral antigens was not performed, histopathological examination of colonic biopsies revealed no amoebae or viral inclusion bodies. Furthermore, the chronic clinical course with markedly elevated fecal calprotectin was not compatible with acute infectious enterocolitis, making these etiologies unlikely. Serum thyroid hormone levels were normal, and there was no history of medication intake that could potentially induce diarrhea. Histopathological findings ruled out conditions such as collagenous colitis and lymphocytic colitis, which are types of microscopic colitis.

## 4. Final Diagnosis

Combined with the patient’s medical history, the patient was finally diagnosed with CD, ileocolonic (L3) type (moderately active).

## 5. Treatment

Treatment based on the Japanese guideline [5] was initiated as remission‐induction therapy with intravenous prednisolone 40 mg/day, administered once daily for six consecutive days. Symptoms began to improve on Day 2, and by Day 6, the patient was well enough for discharge. At discharge, prednisolone was switched to oral 30 mg/day. The dose was then tapered every 2 weeks (30, 20, 15, 10, and 5 mg/day) and discontinued after a total steroid course of approximately 10 weeks. An elemental diet (Elental 80 g/day) was prescribed as concomitant nutritional therapy.

## 6. Outcome and Follow‐Up

Postdischarge, genotyping for the Nudix hydrolase 15 gene confirmed an Arg/Arg variant. Eighteen days after discharge, azathioprine 50 mg/day was initiated as maintenance therapy during the prednisolone taper. After good tolerability had been confirmed over several outpatient visits, the azathioprine dose was titrated to 75 mg/day and later to 100–125 mg/day according to body weight and laboratory monitoring, while prednisolone was being tapered and subsequently discontinued as described above. Apart from azathioprine, the patient continued the elemental diet (Elental 80 g/day) as the only chronic concomitant therapy. Twelve months after the initiation of treatment, the patient remains in clinical remission on azathioprine alone.

## 7. Discussion

We report a case in which a patient, primarily presenting with gastrointestinal symptoms, developed persistent diarrhea for 2 months after SARS‐CoV‐2 infection, leading to a diagnosis of CD. In accordance with the management guidelines for long COVID‐19 by the National Institute for Health and Care Excellence and the Scottish Intercollegiate Guidelines Network, persistent symptoms such as COVID‐19 sequelae are categorized into three phases: acute COVID‐19 (symptoms and findings present up to 4 weeks from onset), ongoing symptomatic COVID‐19 (from 4 to 12 weeks), and post‐COVID‐19 syndrome (symptoms and findings persisting beyond 12 weeks) [6]. In our case, the patient was initially diagnosed with ongoing symptomatic COVID‐19, presenting with prolonged diarrhea.

In a study of 117 COVID‐19 patients hospitalized in China, a 3‐month follow‐up postdischarge revealed that 44% experienced gastrointestinal sequelae, with 17% suffering from chronic diarrhea [3]. Furthermore, a pooled analysis in 2021 found that diarrhea was among the top 10 symptoms of COVID‐19 sequelae, affecting 6% of the patients [4]. These findings suggest that gastrointestinal symptoms are relatively common post‐COVID‐19.

Although gastrointestinal sequelae after COVID‐19 are relatively common, reports of newly diagnosed CD following SARS‐CoV‐2 infection remain scarce, with only a small number of cases described worldwide to date [7–9]. The number of IBD cases triggered by SARS‐CoV‐2 infection remains limited [4]. There have been 10 reported cases of ulcerative colitis [10–13], compared with four reported cases of CD (two adult and two pediatric cases) [7–9]. The first case of CD, reported in July 2020, involved a woman in her 30s diagnosed with COVID‐19 after presenting with fever and upper respiratory symptoms [7]. She initially did not exhibit gastrointestinal symptoms. Although her respiratory symptoms improved quickly, she developed severe watery diarrhea with abdominal pain 1 week later. Following ileocolonoscopy and histopathological examination, she was diagnosed with CD and responded well to treatment with prednisone and sulfasalazine. The second case, reported in May 2021, was a woman in her 40s who presented with fatigue, myalgia, and diarrhea and was diagnosed with COVID‐19 [8]. Her symptoms initially improved within 1 week, but diarrhea recurred and persisted for 4 months. She was eventually diagnosed with CD on the basis of ileocolonoscopy and histopathological examination and responded to treatment with budesonide within 3 weeks. The third and fourth cases of CD [9], reported in April 2023, concern healthy Korean boys (17 and 11 years) who developed severe watery diarrhea, weight loss, and abdominal pain 2–4 weeks after mild SARS‐CoV‐2 infection. Imaging and ileocolonoscopy showed terminal‐ileal/ileocecal ulceration; biopsies revealed noncaseating granulomas or aphthous ulcers, fulfilling Montreal A2 L3 + L4 B1 and A1 L2 B1 criteria. Both improved with steroid induction followed by azathioprine maintenance, extending post‐COVID‐19 de‐novo CD to pediatrics (Table 2). In Japan, the overall prevalence of CD has steadily increased; a recent nationwide survey estimated a prevalence of 77.0 cases per 100,000 population in 2023, with a marked male predominance [14–16]. This highlights that our patient’s demographic profile (young adult male) is consistent with the current epidemiology of CD in Japan, although de novo cases temporally associated with SARS‐CoV‐2 infection remain rare.

**TABLE 2:** Characteristics of patients with Crohn’s disease onset after SARS‐CoV‐2 infection.

Reference number	Sex	Age (years)	COVID‐19 symptoms	COVID‐19 treatment	Time from COVID‐19 onset to CD diagnosis	Type of CD treatment
11	F	33	Sore throat, fever, and myalgia	AcA	1 month	SC and sulfasalazine
12	F	47	Weakness, myalgia, and diarrhea	AcA	4 months	Oral budesonide
13	M	11	Fever	None	A few weeks	SC and azathioprine
13	M	17	Fever, and cough	Antipyretics	2 weeks	SC and azathioprine
Present case	M	18	Fever, diarrhea, and abdominal pain	AcA	2 months	SC and azathioprine

*Note:* SARS‐CoV‐2: severe acute respiratory syndrome coronavirus 2; COVID‐19: coronavirus disease 2019; CD: Crohn’s disease; F: female; M: male; AcA: acetaminophen.

Abbreviation: SC = systemic corticosteroids.

Although the primary symptoms of SARS‐CoV‐2 infection are respiratory related, the occurrence of abdominal symptoms has drawn attention as a potential link between SARS‐CoV‐2 infection and gastrointestinal diseases [17]. It is now known that the receptor for angiotensin‐converting enzyme II, which is highly expressed in gastrointestinal epithelial cells from the esophagus to the rectum, plays a role in the entry of SARS‐CoV‐2 into cells [18]. Additionally, the gastrointestinal system has been implicated in the infection and replication of SARS‐CoV‐2 [19]. Gastrointestinal symptoms can precede respiratory symptoms and, as in the present case, may occur without respiratory manifestations [20].

Regarding IBD, various immunological mechanisms (such as molecular mimicry, epitope spreading, bystander, and polyclonal activation) are involved in the onset and exacerbation of IBD due to numerous pathogens [21]. Anchoring the proposed mechanisms to our case, several features point to barrier dysfunction with postinfectious dysbiosis as the most plausible trigger rather than systemic molecular mimicry. First, gastrointestinal symptoms began concurrently with SARS‐CoV‐2 infection and dominated the clinical picture with minimal respiratory involvement. Second, ileocolonoscopy and biopsy demonstrated patchy terminal‐ileal and right‐colonic disease with longitudinal ulcers and a cobblestone appearance—findings typical of CD. Third, fecal calprotectin was markedly elevated (> 6000 μg/g). SARS‐CoV‐2 enters enterocytes via ACE2 and can replicate in the gut, processes linked to the disruption of epithelial tight junctions and sustained shifts in the intestinal microbiota [18, 19, 22]. Such barrier failure and dysbiosis plausibly amplify innate and adaptive mucosal immune responses (e.g., Th1/Th17) and perpetuate inflammation even after nasopharyngeal viral clearance [21, 22]. In contrast, molecular mimicry would be expected to produce broader or extraintestinal autoimmunity, which was not observed. Bystander activation remains possible; however, in this patient, the chronology and organ specificity support a primary enteric route of immune activation. Collectively, these considerations suggest that SARS‐CoV‐2–induced epithelial injury and dysbiosis, superimposed on host susceptibility, may offer the most parsimonious explanation for de novo CD in this case, although a definitive causal link cannot be established from a single case report. Prior reports of post‐COVID‐19 de novo CD with favorable responses to steroid induction followed by thiopurine maintenance are consistent with this inflammatory mucosal phenotype [7–9].

The current case presented with predominantly gastrointestinal symptoms from the onset of COVID‐19, suggesting possible SARS‐CoV‐2 infection of the intestinal mucosa. After recovery from COVID‐19, the patient experienced temporary relief from abdominal pain, but diarrhea and mild fever persisted, indicating the potential early onset of CD. Elevated fecal calprotectin levels were observed in this case. Fecal calprotectin is a sensitive marker of intestinal mucosal inflammation and is useful in distinguishing inflammatory diseases, such as CD, from functional disorders, such as irritable bowel syndrome. However, fecal calprotectin levels can increase in COVID‐19 patients with persistent diarrhea, regardless of the presence of SARS‐CoV‐2 RNA in the stool [23]. Therefore, this marker is unreliable for differentiating diarrhea related to COVID‐19 from that caused by IBD, and ileocolonoscopy is necessary for the diagnosis.

Given these considerations, it is essential to monitor patients with COVID‐19 who present with gastrointestinal symptoms for the concurrent development of IBD. This is especially important in cases where symptoms such as abdominal pain and diarrhea persist, fever and weight loss are present, gastrointestinal symptoms recur after initial improvement, or fecal calprotectin is elevated. In such cases, it is prudent to consider the onset of IBD as a differential diagnosis and to evaluate its presence or absence by ileocolonoscopy. Our case underscores that persistent diarrhea after SARS‐CoV‐2 infection may reflect new‐onset CD rather than post‐COVID‐19 sequelae alone and that timely ileocolonoscopy is essential for accurate diagnosis.

## Author Contributions

Hironori Yamada contributed to manuscript writing and editing with input from Toru Yamada. Shuji Ouchi and Iichiroh Onishi contributed to manuscript editing and data collection; Ryota Nakamura and Akiko Tamura contributed to manuscript editing; Toru Yamada contributed to writing and editing, conceptualization, and supervision; Masayoshi Hashimoto contributed to manuscript review and editing.

## Funding

No funding was received for this manuscript.

## Disclosure

All authors have read and approved the final manuscript.

## Consent

Written informed consent was obtained from the patient to publish this report in accordance with the journal’s patient consent policy.

## Conflicts of Interest

The authors declare no conflicts of interest.

## Data Availability

The data that support the findings of this study are available from the corresponding author upon reasonable request.

## References

[1] Ng S. C. , Shi H. Y. , Hamidi N. et al., Worldwide Incidence and Prevalence of Inflammatory Bowel Disease in the 21st Century: A Systematic Review of Population-based Studies, The Lancet. (2017) 390, no. 10114, 2769–2778, 10.1016/s0140-6736(17)32448-0, 2-s2.0-85031499214. 29050646

[2] Torres J. , Mehandru S. , Colombel J.-F. , and Peyrin-Biroulet L. , Crohn’s Disease, The Lancet. (2017) 389, no. 10080, 1741–1755, 10.1016/s0140-6736(16)31711-1, 2-s2.0-85007453723. 27914655

[3] Weng J. , Li Y. , Li J. et al., Gastrointestinal Sequelae 90 Days After Discharge for COVID-19, The Lancet Gastroenterology & Hepatology. (2021) 6, no. 5, 344–346, 10.1016/s2468-1253(21)00076-5. 33711290 PMC7943402

[4] Aiyegbusi O. L. , Hughes S. E. , Turner G. et al., Symptoms, Complications and Management of Long COVID: A Review, Journal of the Royal Society of Medicine. (2021) 114, no. 9, 428–442, 10.1177/01410768211032850. 34265229 PMC8450986

[5] Research Group for Intractable Inflammatory Bowel Disease , Rare/Intractable Disease Policy Research Project, Health and Labour Sciences Research Grants, Ministry of Health, Labour and Welfare. Diagnostic Criteria and Treatment Guidelines for Ulcerative Colitis and Crohn’s Disease, 2023, Ministry of Health, Labour and Welfare, Tokyo, https://www.jsibd.jp/wp-content/uploads/2023/07/r4-ibdjapan.pdf.

[6] Shah W. , Hillman T. , Playford E. D. , and Hishmeh L. , Managing the Long Term Effects of COVID-19: Summary of NICE, SIGN, and RCGP Rapid Guideline, BMJ. (2021) 373, no. 136. 10.1136/bmj.n136 33483331

[7] Senthamizhselvan K. , Ramalingam R. , Mohan P. , Kavadichanda C. , Badhe B. , and Hamide A. , De Novo Crohn’s Disease Triggered After COVID-19: Is COVID-19 More than an Infectious Disease?, ACG Case Reports Journal. (2021) 8, no. 8, 10.14309/crj.0000000000000652. PMC8386903 34476279

[8] Tursi A. and Nenna R. , COVID-19 as a Trigger for De Novo Crohn’s Disease, Inflammatory Bowel Diseases. (2022) 28, no. 6, e76–e77, 10.1093/ibd/izab298. 35657373 PMC8690164

[9] Kim K. , Kim S.-Y. , Kim Y. E. , Kwon K.-W. , Han E. M. , and Kim A. , Two Case Reports of Newly Diagnosed Crohn’s disease After COVID-19 in Pediatric Patients, Korean Journal of Gastroenterology. (2023) 81, no. 4, 163–167, 10.4166/kjg.2022.144. PMC12285500 37096436

[10] Tursi A. , Lopetuso L. R. , Vetrone L. M. , Gasbarrini A. , and Papa A. , SARS-CoV-2 Infection as a Potential Trigger Factor for De Novo Occurrence of Inflammatory Bowel Disease, European Journal of Gastroenterology and Hepatology. (2022) 34, no. 8, 883–884, 10.1097/meg.0000000000002379. 35802531 PMC9245529

[11] Preziosi N. A. , Rizvi A. H. , Feerick J. D. , and Mandelia C. , De Novo Pediatric Ulcerative Colitis Triggered by SARS-CoV-2 infection: A Tale of 2 Sisters, Inflammatory Bowel Diseases. (2022) 28, no. 10, 1623–1625, 10.1093/ibd/izac142. 35762665 PMC9384334

[12] Morita A. , Imagawa K. , Tagawa M. , Sakamoto N. , and Takada H. , Case Report: Immunological Characteristics of De Novo Ulcerative Colitis in a Child Post COVID-19, Frontiers in Immunology. (2023) 14, 10.3389/fimmu.2023.1107808. PMC9978098 36875135

[13] Swatski M. D. , Kaur P. , Borlack R. E. , McBain S. , Uffer J. , and Almadhoun O. , A Case Series of New-Onset Ulcerative Colitis Following Recent Diagnosis of COVID-19, JPGN Rep. (2023) 4, no. 4, 10.1097/pg9.0000000000000383. PMC10684170 38034458

[14] Chiba M. , Morita N. , Nakamura A. , Tsuji K. , and Harashima E. , Increased Incidence of Inflammatory Bowel Disease in Association with Dietary Transition (Westernization) in Japan, JMA Journal. (2021) 4, no. 4, 347–357, 10.31662/jmaj.2021-0038. 34796289 PMC8580716

[15] Tsutsui A. , Murakami Y. , Nishiwaki Y. et al., Nationwide Estimates of Patient Numbers and Prevalence Rates of Ulcerative Colitis and Crohn’s Disease in Japan in 2023, Journal of Gastroenterology. (2025) 60, no. 12, 1513–1522, 10.1007/s00535-025-02295-z. 40892110

[16] Zhang Y. , Chung H. , Fang Q.-W. et al., Current and Forecasted 10-year Prevalence and Incidence of Inflammatory Bowel Disease in Hong Kong, Japan, and the United States, World Journal of Gastroenterology. (2025) 31, no. 18, 10.3748/wjg.v31.i18.105472. PMC12146929 40496360

[17] Lewandowski K. , Kaniewska M. , Rosolowski M. , and Rydzewska G. , Gastrointestinal Symptoms in COVID-19, Przegląd Gastroenterologiczny. (2023) 18, no. 1, 61–66. 10.5114/pg.2021.112683 PMC10050985 37007763

[18] Zou X. , Chen K. , Zou J. , Han P. , Hao J. , and Han Z. , Single-Cell RNA-Seq Data Analysis on the Receptor ACE2 Expression Reveals the Potential Risk of Different Human Organs Vulnerable to 2019-nCoV Infection, Frontiers of Medicine. (2020) 14, no. 2, 185–192, 10.1007/s11684-020-0754-0. 32170560 PMC7088738

[19] Parasa S. , Desai M. , Chandrasekar V. T. et al., Prevalence of Gastrointestinal Symptoms and Fecal Viral Shedding in Patients with Coronavirus Disease 2019, JAMA Network Open. (2020) 3, no. 6, 10.1001/jamanetworkopen.2020.11335. PMC7290409 32525549

[20] Luo S. , Zhang X. , and Xu H. , Don’T Overlook Digestive Symptoms in Patients with 2019 Novel Coronavirus Disease (COVID-19), Clinical Gastroenterology and Hepatology. (2020) 18, no. 7, 1636–1637, 10.1016/j.cgh.2020.03.043. 32205220 PMC7154217

[21] Lidar M. , Langevitz P. , and Shoenfeld Y. , The Role of Infection in Inflammatory Bowel Disease: Initiation, Exacerbation and Protection, The Israel Medical Association Journal. (2009) 11, no. 9, 558–563. 19960852

[22] Zuo T. , Zhang F. , Lui G. C. et al., Alterations in Gut Microbiota of Patients with COVID-19 During Time of Hospitalization, Gastroenterology. (2020) 159, no. 3, 944–955, 10.1053/j.gastro.2020.05.048. 32442562 PMC7237927

[23] Effenberger M. , Grabherr F. , Mayr L. et al., Faecal Calprotectin Indicates Intestinal Inflammation in COVID-19, Gut. (2020) 69, no. 8, 1543–1544, 10.1136/gutjnl-2020-321388. 32312790 PMC7211078

